# Cochlear Implants in Single-Sided Deafness. Comparison Between Children and Adult Populations With Post-lingually Acquired Severe to Profound Hearing Loss

**DOI:** 10.3389/fneur.2021.760831

**Published:** 2021-11-04

**Authors:** Nadia Falcón Benítez, Juan Carlos Falcón González, Ángel Ramos Macías, Silvia Borkoski Barreiro, Ángel Ramos de Miguel

**Affiliations:** ^1^Department of Clinical Sciences, University of Las Palmas de Gran Canaria, Las Palmas, Spain; ^2^Department of Otolaryngology, Head and Neck Surgery, Complejo Hospitalario Universitario Insular Materno Infantil de Gran Canaria, Las Palmas, Spain; ^3^Hearing and Balance Laboratory, Las Palmas de Gran Canaria University Institute of Intelligent System and Numeric Application in Engineering, Las Palmas, Spain

**Keywords:** cochlear implant, single-sided deafness, hearing loss, sound localization, spatial listening

## Abstract

**Objective:** To determine audiological and clinical results of cochlear implantation (CI) comparing two populations with single-sided deafness (SSD): post-lingually deaf children between 6 and 12 years of age, and post-lingually deaf adults, in order to evaluate the effect of CI in different age groups.

**Design:** Retrospective case review.

**Setting:** Tertiary clinic.

**Patients and Method:** Twenty-three children and twenty-one adult patients that were candidates for CI with single-side deafness were included. In all cases we evaluate: Speech perception thresholds; disyllabic words test (65 dB SPL) were performed in the modalities S0–SCI–SNH and Auditory Lateralization Test. The Speech, Spatial, and Qualities of Hearing Scale (SSQ) questionnaire was also used. All results were obtained after 12 months of CI activation.

**Results:** In children, the most common etiology was idiopathic sensory-neural hearing loss. They showed positive results in the Auditory Lateralization Test. In the Speech Test, word recognition in noise improved from 2% preoperatively to 61.1% at a mean follow-up of 1 year (S0 condition) in children [test with signal in CI side 60% and signal normal hearing side (plugged) 31%]. The processor was used for >12 h in all cases. With respect to the SSQ questionnaire, parents were more satisfied within the postoperative period than within the preoperative period. For adults, the most common etiology was idiopathic sudden sensorineural hearing loss (SNHL). Positive results in the Auditory Lateralization Test were found. With respect to the Speech Test in quiet conditions: Word recognition in noise improved from 5.7% preoperatively to 71.8% at a mean follow-up of 1 year [test with signal in CI side 68% and signal normal hearing side (plugged) 41%]. The processor was used for >12 h. In the SSQ questionnaire, the post-operative results showed a beneficial effect of the CI. No adverse events were reported during the study period. No differences were found between children and adults in all tests in this study.

**Conclusions:** Cochlear implantation in post-lingually deaf adults and children with SSD can achieve a speech perception outcome comparable with CI in conventional candidates. Improvements in spatial hearing were also observed. Careful patient selection and counseling regarding potential benefits are important to optimize outcomes.

## Introduction

Binaural hearing allows human beings to perform effective communication. Thus, single-sided deafness (SSD) leads to relevant hearing difficulties in most daily situations (1). Single-sided deafness affects sound localization, speech comprehension in noisy environments, spatial awareness, hearing easiness, and spoken language development. Back in the 1960s, Giolas and Wrak suggested that these difficulties and their consequences on vocational and social activities can cause discomfort, shame, and impotency feelings (2).

Cochlear implantation (CI) for single-sided deafness was firstly considered as a treatment to suppress severe tinnitus in adults, and, shortly after, binaural hearing re-establishment was considered as another benefit of this implantation on the single-sided hearing loss (3–5). CI as a beneficial treatment for adults suffering from acquired SSD is well-established in a growing number of countries, but there is little experience about this treatment option in children (6–11).

The mechanisms by which single-sided deafness affects language, and academic and cognitive performance are related to impaired spatial capabilities and to binaural audition. Children with congenital SSD show a significant audiological and subjective improvement when they are treated with CI at an early age. In addition, children with post-lingual SSD and a short period of hearing deprivation are able to integrate their normal acoustic hearing with the electrical signal of the cochlear implant and they show binaural improvement (10, 12, 13). In adults with post-lingual single-sided deafness, there is evidence of binaural function restoration after cochlear implantation (14).

The aim of the present study is to determine audiological and clinical results of CI comparing two populations with SSD: post-lingually deaf children between 6 and 12 years of age, and adults, in order to evaluate the effect of CI in different age groups.

## Materials and Methods

This is an observational, descriptive, transversal study performed at Complejo Hospitalario Universitario Insular Materno Infantil de Gran Canaria, Hipoacusia Unit, Dept. Otolaryngology. conducted in adults, and children below 12 years of age, with acquired SSD, who received CI between October 2019 and May 2020 in our department, with a minimum follow-up of 12 months.

In all cases, there were no implanted patients with ossification or any other cochlear anomalies that might prevent complete insertion of the electrode array; severe to profound hearing loss related to meningitis, multiple sclerosis, posterior fossa tumors, or central hearing related disorders; signs of retro-cochlear or central origin of hearing impairment; medical conditions that would contraindicate undergoing CI surgery (e.g., active middle ear infections, tympanic membrane perforation); psychological, neural, or mental disorders that would contraindicate undergoing CI surgery as verified by a psychologist; or any other additional handicaps that world prevent participation in evaluations.

The cochlear implants used in this study were the Nucleus® Profile with Slim Electrode Modiolar CI632, and the Nucleus® CI612.

The following presurgical tests were performed:

- Auditory Steady-State Responses (ASSR).
- Pure Tone Audiometry (PTA).
- Transient Evoked Otoacoustic Emissions (TEOAE).
- Cerebral Magnetic Resonance Imaging (MRI), and High-resolution computer tomography or Cone Beam CT.
- Genetic testing (Conexin 26, otoferlin).
- Speech Test in quiet and noise settings.

All subjects and their legal guardians in the case of children were informed about the benefits and disadvantages of the possible treatment options for single-sided deafness (no treatment, CROS system, bone-conduction system, or CI). The cochlear implantation expectations were also adjusted. Before the surgery, patients tested a bone-conduction device and the CROS system device.

The post-surgical tests were performed after 12-months experience in the use of their respective speech processor. For the Speech Test, protocol for the assessment of hearing in the Spanish language and its version adapted to the infant population was used (15). The test was conducted in the modalities azimuth (S0), signal CI side (SCI), and signal normal hearing side (SNH), the normal-hearing ear was masked by an auditory threshold for white noise of +10 dB and a complete plugging and noise-canceling earphone.

In both groups, the TEOAE and ASSR responses were analyzed by the Eclipse Interacoustics Modules with the OtoAcces® Database.

In the children group, cortical response was analyzed using the Hear System EARLab™ Aided Cortical Assessment (ACA) 1.0, using the stimuli “m,” “t,” and “s.”

To perform the Lateralization Test on both groups, five speakers were used in positions 0°, 45°, 90°, 270°, and 370°, and 1,000 and 2,000 Hz pure tones were used at 65 dB. Signals were presented randomly 10 times per subject, and the test was considered positive when the success rate was ≥ 80%.

All the tests were conducted in soundproof cabins (two connected soundproof cabins: one for each subject and the other for the operator), and by using an Audiotest 340 Interacoustics AS DK-5610 Assens. Denmark 2008 CE 0123 audiometer and Resolv Active Studio Monitor A5 45Hz- 27 Khz Biamped 50 watt speakers.

A questionnaire measured the postoperative change in the parent's ratings of the child's performance in specific listening situations. Items were related to speech perception, spatial hearing, or other qualities of hearing (Speech, Spatial, and Qualities of Hearing Scale, SSQ). Regular reports about the device use, attitude, and performance were taken from adults and from parents (16, 17).

Data analysis was performed using SPSS 25.0 (18). Within the different groups, the categorical variables were expressed in percentages and absolute frequencies, while numerical variables were expressed as average, median, and standard deviation values. Percentages were compared by using the Chi-square test, and average values were compared by using the Student *t*-test for paired samples. ANOVA or the non-parametric Kruskal–Wallis test for independent samples were used to compare average and median values in more than two groups. Statistical significance was set at *P* < 0.05.

The study obtained the approval of the Ethical Committee of our hospital in accordance with the Declaration of Helsinki. All adults and parents or legal guardians of the included participants provided written consent information.

## Results

Forty-four cochlear implant users diagnosed with SSD were studied between June 2019 and February 2020. The children sample consisted of 29 subjects: 6 of which (20.69%) were non-implanted subjects because they presented congenital malformations: 3 had auditory nerve agenesis, 2 had cases of major cochlear malformation (common cavity), and 1 had a case of cochlear agenesis; and 23 of which (52.3%) had been implanted. The 23 patients in the implanted children group consisted of 10 boys (43.5%) and 13 girls (56.5%). With respect to the adult group, they were 21 patients (47.7%): 7 men (33.3%) and 14 women (66.7%).

The average age of the children was 7.15 years with an SD of 1.46 years, while the adults' average age was 49.47 years (SD = 8.80). The average age of the whole sample was 27.35 years (SD = 22.23).

With respect to the causes of SSD in children, their etiologies were progressive hearing loss of unknown origin in 17 subjects (73.91%), a sudden hearing loss in 4 subjects (17.39%), and cholesteatoma in 2 subjects (8.70%). With respect to the causes of SSD in adults, their etiologies were progressive hearing loss of unknown origin in 12 subjects (57.14%), sudden hearing loss in 4 subjects (19.05%), Meniere's disease in 3 subjects (14.29%), and acoustic trauma in 2 subjects (9.52%). The presurgical average PTA of the whole sample was 84 dB ±12 in the implanted ear and 28 dB ±4 in the normal-hearing ear.

In Tables 1, 2, the characteristics and results of the evaluations performed on children and adults, respectively, can be observed. The Lateralization Test showed results in both groups for the 0°, 45°, 90°, 270°, and 315° modalities. In the children group, cortical responses in the auditory association areas were registered after the presented stimuli. Statistically significant differences were obtained when comparing the hearing deprivation period between the two studied groups (*p* = 0.04), being 12.13 months (SD = 4.52) in children and 9.85 months (SD = 2.41) in adults. While no significant differences were obtained when comparing the cochlear implantation periods (*p* > 0.05) of 17.61 months (SD = 5.76) in children and 15.14 months (SD = 3.26) in adults. With respect to the everyday use of the processor, the average use value was 10.16 h (range 7–13).

**Table 1:** Characteristics and results of children the single-sided deafness children group.

**Sub**.	**Sex**	**Age** **years/months**	**Etiology**	**CI** **ear**	**Hearing depriv. months**	**CI** **type**	**CI** **use** **months**	**CI** **use** **hour/day**	**Disyllabic word score**	**Masking umbral** **+10 WN**			**Lateralization**				**Cortical** **resp.** **m, t, s**
										**Signal** **Azimuth**	**Signal CI** **side**	**Signal NH** **side**	**Signal CI side** **45^**°**^**	**Signal Ci side** **90^**°**^**	**Signal NH** **270^**°**^**	**Signal NH** **315^**°**^**	
										**w CI**	**w CI**	**w CI**					
1	F	7.1	Sudden HL	L	18	612	30	11	96	68	68	60	+	+	+	–	Positive
2	F	6	Unknown	L	6	632	12	13	100	60	64	52	+	+	+	–	Positive
3	M	12	Cholesteatoma	L	24	612	24	12	100	56	64	52	+	+	+	+	Positive
4	F	6.2	Unknown	L	12	632	12	10	96	52	64	48	+	+	+	+	Positive
5	M	6.3	Unknown	R	12	632	18	10	100	52	56	44	–	+	+	+	Positive
6	F	6.2	Unknown	L	6	632	24	9	100	56	68	40	+	+	+	+	Positive
7	F	6.7	Unknown	L	9	632	12	10	96	52	56	48	+	+	+	–	Positive
8	M	6.1	Sudden HL	R	6	632	12	12	96	48	60	52	+	+	+	+	Positive
9	M	6.2	Unknown	L	18	612	30	13	96	60	64	48	+	+	+	+	Positive
10	M	8.1	Unknown	R	14	612	14	12	100	60	64	52	–	+	+	+	Positive
11	F	7.1	Unknown	R	8	632	16	12	100	52	56	44	+	+	+	+	Positive
12	M	6.2	Unknown	L	16	632	24	11	100	52	56	48	+	+	+	+	Positive
13	F	10.2	Sudden HL	R	12	612	13	10	96	52	52	44	–	+	+	+	Positive
14	F	6.7	Unknown	R	11	632	14	11	100	60	64	48	+	+	+	+	Positive
15	M	8.4	Unknown	L	10	612	15	9	96	56	60	48	+	+	+	-	Positive
16	M	7.2	Unknown	L	10	632	17	13	96	60	68	52	+	+	+	+	Positive
17	F	6.4	Sudden HL	R	12	632	16	9	100	48	52	44	–	+	+	+	Positive
18	F	6.7	Unknown	L	15	632	22	10	100	52	56	44	+	+	+	+	Positive
19	F	6	Unknown	R	14	632	13	12	92	60	64	48	–	+	+	+	Positive
20	F	6.1	Unknown	L	6	612	14	11	100	60	64	52	+	+	+	-	Positive
21	F	7.3	Unknown	R	14	632	14	10	100	60	64	52	+	+	+	+	Positive
22	M	7	Unknown	L	16	612	24	11	100	52	56	48	+	+	+	–	Positive
23	M	8.2	Cholesteatoma	R	10	612	15	11	96	56	60	48	+	+	+	+	Positive

**Table 2:** Characteristics and results of adults single-sided deafness adult group.

**Sub**.	**Sex**	**Age years/** **months**	**Etiology**	**CI ear**	**Hearing depriv. months**	**CI type**	**CI use months**	**CI use hour/** **day**	**Disyllabic word score (%)**	**Masking umbral** **+10 WN**			**Lateralization**			
										**Signal** **Azimuth**	**Signal CI** **side**	**Signal NH** **side**	**Signal CI** **side** **45^**°**^**	**Signal Ci** **side** **90^**°**^**	**Signal NH** **270^**°**^**	**Signal NH** **315^**°**^**
										**w CI (%)**	**w CI (%)**	**w CI (%)**				
1	M	35.8	Sudden HL	R	18	632	18	10	96	68	68	60	–	+	+	+
2	F	58.7	Unknown	L	6	612	14	7	100	60	64	52	+	+	+	+
3	M	47.7	Unknown	L	24	632	12	9	100	56	64	52	+	+	+	-
4	F	59	Unknown	R	12	612	12	12	96	52	64	48	+	+	+	+
5	M	57.4	Sudden HL	R	12	612	11	9	100	52	56	44	–	+	+	+
6	M	38.4	Sudden HL	L	6	632	10	10	100	56	68	48	+	+	+	+
7	F	49.6	Unknown	L	9	632	12	11	92	56	64	48	+	+	+	-
8	F	47.5	Sudden HL	R	6	632	13	11	96	60	68	52	+	+	+	+
9	M	48.5	Unknown	L	18	632	16	9	96	60	64	48	+	+	+	–
10	F	49.9	Unknown	R	14	612	18	10	100	60	64	52	-	+	+	+
11	F	56.4	Menière	L	8	632	18	10	100	52	56	44	+	+	+	–
12	M	58.4	Acoustic trauma	L	16	612	20	10	100	52	56	48	+	+	+	+
13	F	59	Acoustic trauma	R	12	612	20	8	96	60	52	52	+	+	+	+
14	F	55.4	Unknown	L	11	612	20	7	100	56	64	52	+	+	+	+
15	M	44.8	Unknown	L	10	632	18	9	96	64	60	60	+	+	+	-
16	F	37.4	Unknown	L	10	612	14	10	96	56	68	52	+	+	+	–
17	F	35.2	Unknown	R	12	612	12	11	100	60	52	52	+	+	+	+
18	M	34.8	Menière	L	15	632	13	9	100	68	56	48	+	+	+	–
19	F	48.6	Unknown	L	14	632	13	8	96	52	64	48	+	+	+	+
20	F	58.7	Unknown	L	6	612	16	8	92	60	64	52	+	+	+	+
21	F	57.6	Menière	R	14	612	18	7	92	56	64	52	–	+	+	+

**Figure 1:**
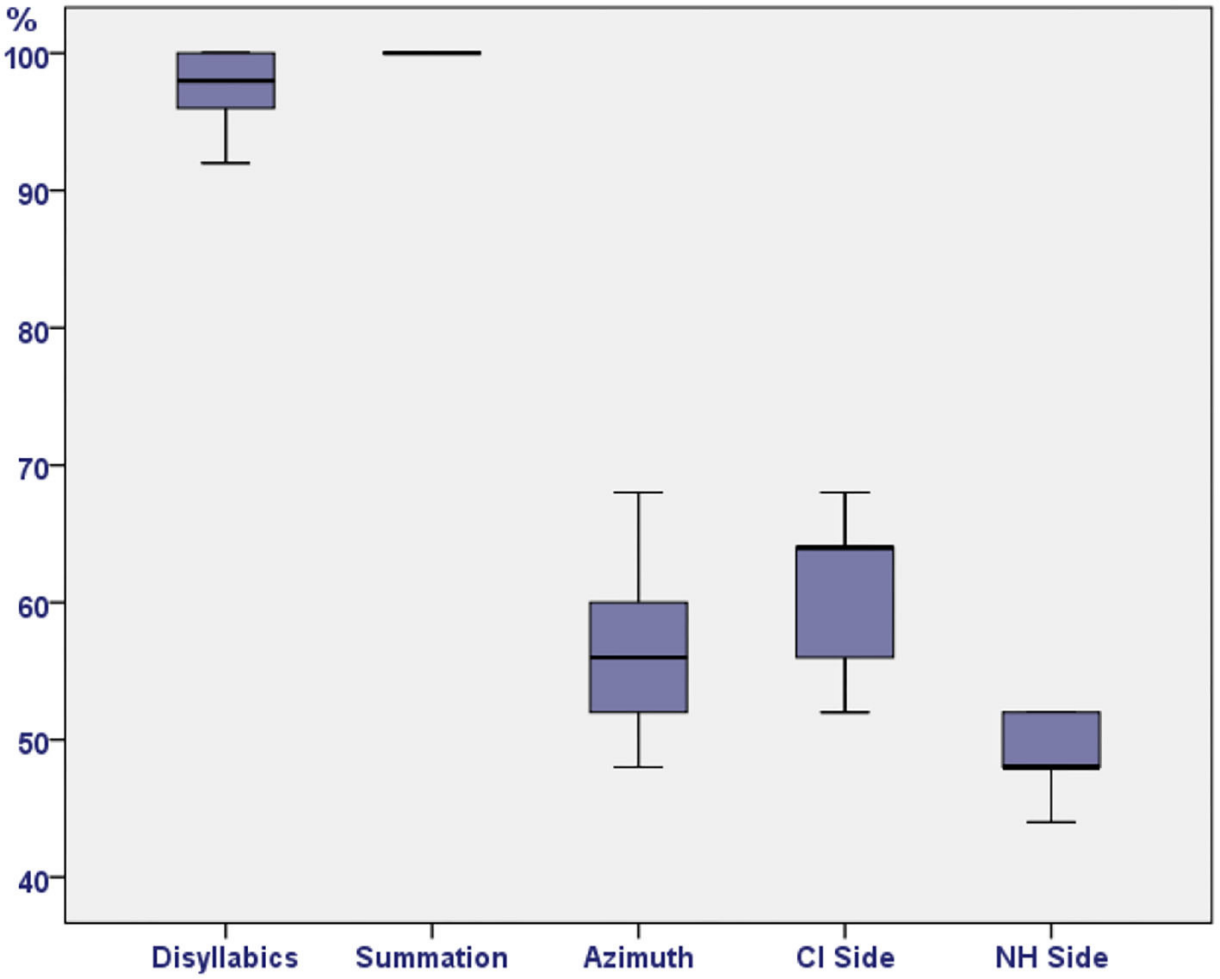
Acquired single-sided deafness: speech test results.

Discrimination in the Speech Test reached recognition values of 92 and 100%; it was observed that all the subjects reached 100% recognition if the summation effect was considered. The results obtained in the S0 modality (azimuth) were 48 and 68% (mean 56.55%) in discrimination, the SCI modality ranged from 52 to 68% (mean 61.36%), and, in the SNH modality, the recognition percentage ranged from 44 to 52% (mean 49.09%; Table 3 and Figure 1).

**Table 3:** Acquired single-sided deafness: speech test results.

	**Children (*****n*** **=** **23)**				**Adults (*****n*** **=** **21)**			
	**Min. %**	**Max. %**	**MD %**	**SD**	**Min. %**	**Max. %**	**MD %**	**SD**
Disyllabics without CI in quiet	92	100	97.57	2.889	92	100	97.33	2.921
Disyllabics without CI in noise	84	96	92.16	3.787	88	96	92.48	3.331
Azimuth 0°	48	68	55.83	4.896	52	64	57.33	3.864
Signal CI side	52	68	60.87	4.966	52	68	61.90	5.157
Signal NH side	44	52	48.35	3.171	44	52	49.90	2.719

When the results of the three SSQ questionnaire subscales studied were analyzed (range 1–10), in both groups, the adults' satisfaction within the post-surgical period ranged from 7 to 10, while the parents' satisfaction within the postoperative period reached 8 to 10, which were higher than those obtained for the preoperative period.

**Figure 2:**
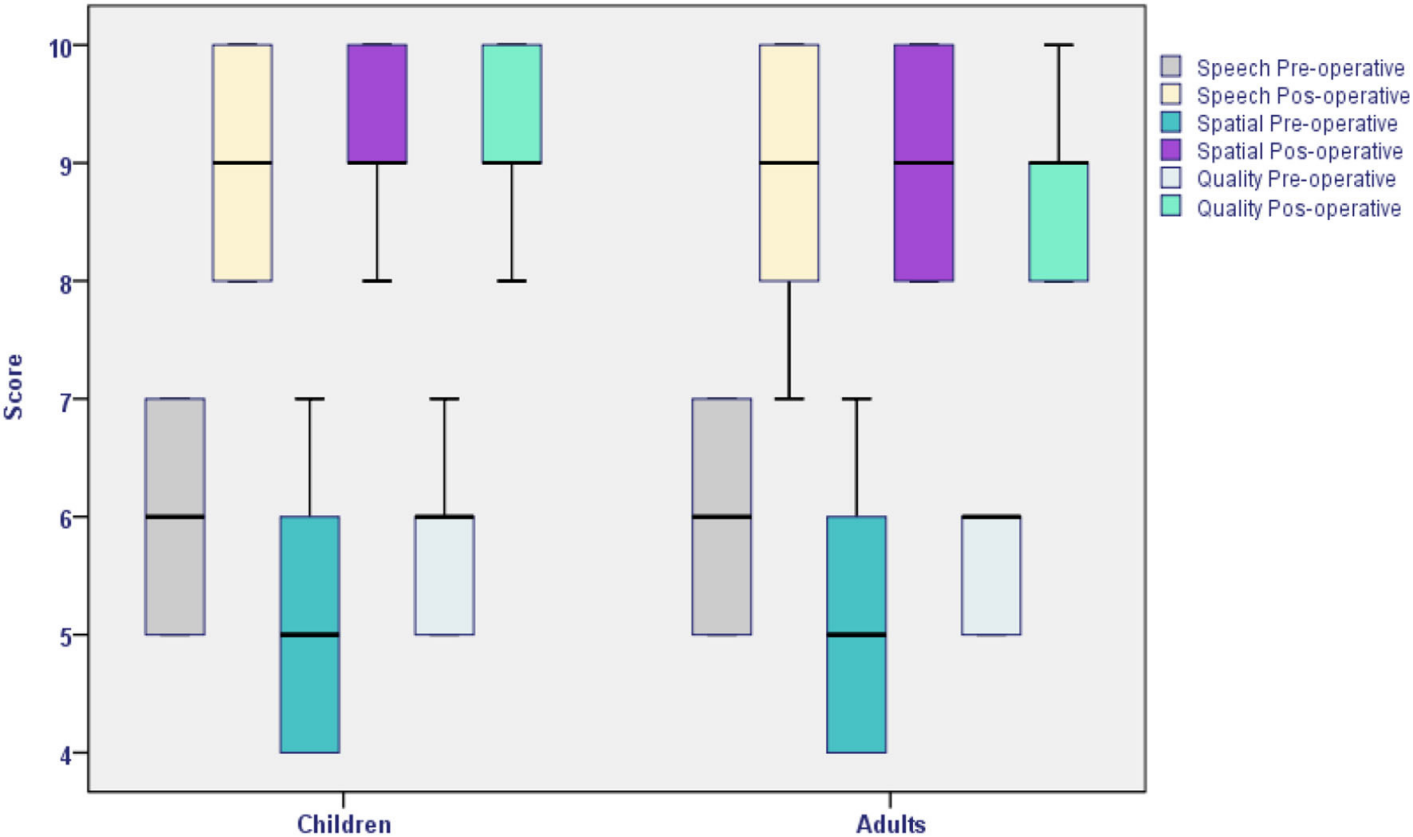
Speech, Spatial, and Qualities of Hearing Scale questionnaire results of the children and adult single-sided deafness groups (*p* > 0.05).

Two independent sample tests were used to compare the results of both groups: The Levene test for equality of variances and the Student *t*-test for equality of means. No statistically significant differences were obtained (*p* > 0.05; Table 4 and Figure 2).

**Table 4:** Speech, Spatial, and Qualities of Hearing Scale questionnaire results of the children and adult single-sided deafness groups (*p* > 0.05).

	**Children (*****n*** **=** **23)**				**Adults (*****n*** **=** **21)**			
	**Min**.	**Max**.	**MD**	**SD**	**Min**.	**Max**.	**MD**	**SD**
Speech pre-operative	5	7	6.09	5	5	7	6.10	0.889
Speech post-operative	8	10	8.87	8	7	10	8.76	1.136
Spatial pre- operative	4	7	5.00	4	4	7	4.90	0.995
Spatial post-operative	8	10	9.22	8	8	10	9.10	0.831
Quality pre-operative	5	7	5.83	5	5	6	5.57	0.507
Quality post-operative	8	10	9.17	8	8	10	8.81	0.814

## Discussion

Bilateral and binaural hearing make efficient communication possible. A decrease in peripheral auditory input and a lack of binaural summation lead to the loss of relevant acoustic information, which affects sound localization, speech comprehension in noisy environments, spatial awareness, hearing easiness, and spoken language development (12, 19, 20). Several authors have described changes within the central nervous system in adults and children suffering from single-sided hearing loss. These alterations include auditory structures and/or other brain structures and are due to an impaired auditory input (21–23).

These alterations affect auditory and neurocognitive factors. That is the reason by which CI may be the only treatment to provide their users with useful hearing information, especially at the pediatric age. Without this support, little or no information can be received, so CI improves general communication (12). As hearing remains active in one of the ears, this ear maintains the sound frequency representation in the brain's auditory cortex of the “deaf” side. After CI, the brain can use these conserved cortical ways and representations to process and interpret the sounds coming from the cochlear implant, which facilitates hearing restoration.

Wedekind et al. (24) evidenced in their study that brains can interpret the signals coming from cochlear implants in an independent manner, by performing processes that are similar to those observed in normal hearing. In accordance with these authors, when cortical potentials were applied on the implanted ear of the subjects of this study, the registered latencies of their auditory cortex evoked responses that maintained the same structure of those from normal hearing ears (24).

In relation to the programming strategy, the Frequency-Allocation Programming (FAP) method was then used in our patients. This method permits intensity level decreases and dynamic range increases, so it decreases the overlapping band while mapping and improves the audio quality of the signal representation (25).

One of the most frequently used tools to measure the quality of life related to hearing is the Speech, Spatial, and Qualities of Hearing Scale (SSQ). In this study, significant benefits in the determinations of quality of life related to hearing were observed in both groups: by adults and children's parents.

Several studies on adults with SSD have described better sound localization, speech comprehension in noisy environments, and decrease of tinnitus severity after cochlear implantation. In this study, a better sound localization was confirmed and, similarly to what was described by Hwa et al., an excellent speech comprehension after cochlear implantation was demonstrated (4, 26–28).

One of the indirect signals to assess the real benefit of CI in children is the time of use of their speech processor. Motivated adults and children suffering from SSD but with good expectations can benefit from cochlear implantation, and it has been observed that most of them continue to be full-time cochlear implant users. Even when patients with bilateral deafness that have a cochlear implant show a decrease in the time of use of their speech processor, this decrease is generally due to a lack of objective hearing benefit and/or to social stigma. These aspects are important for the pediatric population of cochlear implant users (29, 30).

In our study, children used their sound processor on a full-time basis and showed a good acceptation of the devices; these results are similar to those described by Ganek et al., who did not observe significant changes in the use of cochlear implants as the children grew up or acquired more hearing experience. Differently, Greaver et al. observed variability of the speech processor time of use in their study conducted on children (31, 32).

A high incidence of auditory nerve agenesis within the population of children suffering from SSD was observed in this study, similar to that described in previous studies (33–35).

## Conclusions

Cochlear implantation in post-lingually deaf adults and children with SSD can achieve a speech perception outcome comparable with CI in conventional candidates. Improvements in spatial hearing were also observed. Careful patient selection and counseling regarding potential benefits are important to optimize outcomes, mainly in children with acquired SSD and those implanted after a longer period that may not have experienced a significant benefit (binaural), although other bilateral effects can be achieved.

Taken together with other studies, this research enables doctors to take evidence-based clinical decisions about how to manage single-sided deafness in both children and adult groups. To obtain successful results, it must be considered that a rigorous selection of candidates and a proper adjustment between clinical advice and expectations are essential.

## Data Availability Statement

The raw data supporting the conclusions of this article will be made available by the authors, without undue reservation.

## Ethics Statement

The studies involving human participants were reviewed and approved by Comité de Ética de la Investigación/Comité de Ética de la Investigación con Medicamentos Hospital Universitario de Gran Canaria Dr. Negrín (CEI/CEIm HUGCDN). Written informed consent to participate in this study was provided by the participants' legal guardian/next of kin.

## Author Contributions

NF: data acquisition and manuscript writing. JF: data acquisition, verification and patients' fitting, and intraoperative testing. ÁRMa: original idea, manuscript writing, and surgeries. SB: manuscript and patients' agenda control. ÁRMi: manuscript and intraoperative testing. All authors contributed to the article and approved the submitted version.

## Conflict of Interest

The authors declare that the research was conducted in the absence of any commercial or financial relationships that could be construed as a potential conflict of interest.

## Publisher's Note

All claims expressed in this article are solely those of the authors and do not necessarily represent those of their affiliated organizations, or those of the publisher, the editors and the reviewers. Any product that may be evaluated in this article, or claim that may be made by its manufacturer, is not guaranteed or endorsed by the publisher.
